# Dental caries incidence in infants with abnormal birth weight for gestational age: a longitudinal study in the Japan Environment and Children’s Study

**DOI:** 10.1265/ehpm.26-00133

**Published:** 2026-09-30

**Authors:** Masahiro Tsuchiya, Shinobu Tsuchiya, Haruki Momma, Jun Aida, Masahiro Iikubo, Yuichiro Miura, Chiharu Ota, Japan Environment and Children’s Study Group

**Affiliations:** 1 Department of Nursing, Tohoku Fukushi University, Sendai, Miyagi, Japan; 2 Department of Orthodontics and Speech Therapy for Craniofacial Anomalies, Tohoku University Hospital, Sendai, Miyagi, Japan; 3 Division of Craniofacial Anomalies, Tohoku University Graduate School of Dentistry; 4 Laboratory of Well-Being, Center for Physical Activity Research, National Institute of Health and Nutrition, National Institutes of Biomedical Innovation, Health and Nutrition, Settsu, Osaka, Japan; 5 Department of Dental Public Health, Graduate School of Medical and Dental Sciences, Institute of Science Tokyo, Tokyo, Japan; 6 Division of Dental Informatics and Radiology, Graduate School of Dentistry, Tohoku University, Sendai, Japan; 7 Department of Feto-Maternal Medical Science, Tohoku University Graduate School of Medicine, Sendai, Miyagi, Japan; 8 Environment and Genome Research Center, Tohoku University Graduate School of Medicine, Sendai, Japan; 9 Department of Informative Genetics, Environment and Genome Research Center, Tohoku University Graduate School of Medicine

**Keywords:** Dental caries, Birth weight for gestational age, A longitudinal study, Birth cohort

## Abstract

**Background:**

Early childhood caries (ECC) remains a major global public health concern, influenced by complex socioeconomic and biological factors. Although deviations in fetal growth, including being small for gestational age (SGA) or large for gestational age (LGA), have been linked to various long-term health outcomes, evidence regarding their association with ECC remains inconsistent. This study examined the influence of SGA and LGA on the cumulative experience of ECC and severe ECC (S-ECC) using a large-scale nationwide birth cohort.

**Methods:**

This prospective cohort study utilized data from the Japan Environment and Children’s Study, a nationwide investigation of approximately 100,000 mother–child dyads. After applying exclusion criteria, 79,006 children were included in the final analysis. SGA and LGA were defined according to Japanese reference standards. Information on ECC experience was obtained from caregiver-reported questionnaires administered at 4 and 5 years postpartum. Based on established criteria, S-ECC was defined as any caries experience by 3 years of age, and ECC as any experience by 5 years of age. A generalized linear mixed model (GLMM) was used to estimate risk ratios (RRs) and 95% confidence intervals (CIs) for longitudinal ECC trajectories. Sensitivity analyses using Poisson regression with multiple imputation were conducted to assess the robustness of findings.

**Results:**

Longitudinal trajectories demonstrated distinct differences in ECC risk across the birth weight spectrum. In fully adjusted GLMMs, children born LGA had significantly higher risks of both S-ECC and ECC than those born appropriate for gestational age (S-ECC: RR = 1.13, 95% CI: 1.08–1.18; ECC: RR = 1.13, 95% CI: 1.10–1.15). In contrast, children born SGA exhibited significantly lower risks (S-ECC: RR = 0.91, 95% CI: 0.86–0.96; ECC: RR = 0.94, 95% CI: 0.92–0.97). These associations remained consistent in sensitivity analyses addressing missing data.

**Conclusions:**

Children born LGA demonstrated a relatively higher prevalence of ECC, consistent with findings from previous studies. Conversely, the lower prevalence observed among children born SGA may reflect greater parental vigilance or enhanced medical monitoring. Birth weight for gestational age may therefore serve as a useful indicator for identifying children who could benefit from targeted oral health interventions.

**Supplementary information:**

The online version contains supplementary material available at https://doi.org/10.1265/ehpm.26-00133.

## Graphical Abstract

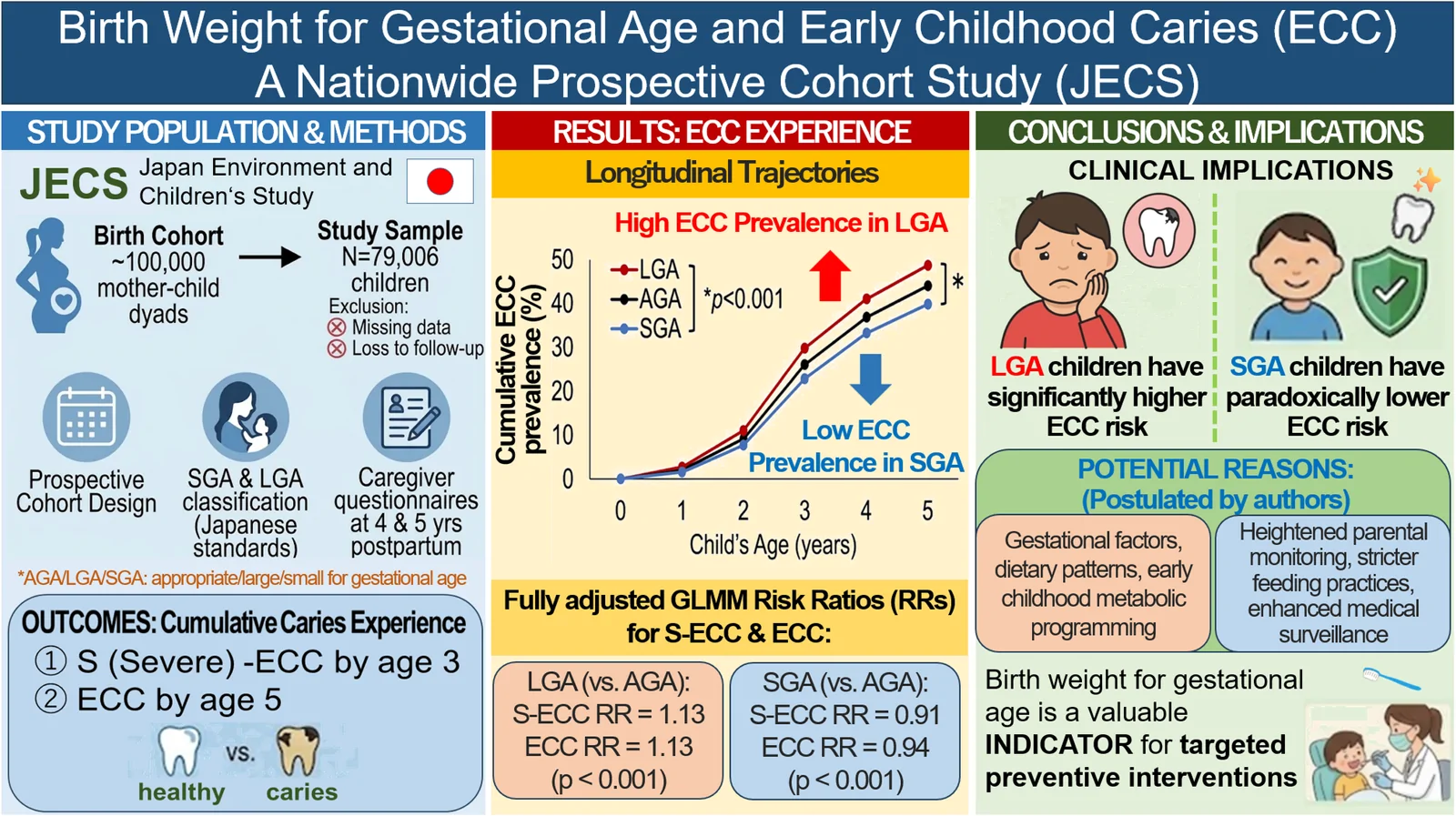

## Background

Early childhood caries (ECC), defined as the presence of one or more decayed, missing, or filled tooth surfaces in children under 6 years of age, adversely affects quality of life through pain, infection, and potential developmental complications [1, 2]. Furthermore, the presence of any sign of dental caries in a child younger than 3 years is classified as severe ECC (S-ECC), representing an advanced form of the disease [3]. Despite advances in preventive and clinical care, the 2021 Global Burden of Diseases Study reported little change in the global prevalence and burden of ECC between 1990 and 2021, particularly in low- and middle-income countries [4, 5]. Although overall oral health has improved in Japan, ECC remains a public health concern, with recent reports showing prevalence rates of 0.6% among 18-month-olds and 7.4% among 3-year-olds [6]. Twice-daily toothbrushing with fluoride toothpaste is strongly recommended as a primary preventive measure [7, 8]. However, because ECC is a biofilm-mediated, sugar-driven disease [1–5], parental health management and caregiving practices remain critical to its prevention.

Socioeconomic factors, including family income, parental education, occupation, and living conditions, substantially influence children’s oral health outcomes [9, 10]. Furthermore, recent studies have reported an increased risk of dental caries among children with abnormal birth weight or gestational age [3, 11–15], factors that are also linked to neonatal morbidity and the later development of noncommunicable diseases owing to metabolic dysfunction, characterized by impaired vascular and endocrine homeostasis [16–20]. Based on sex-specific standardized growth charts, small-for-gestational-age (SGA) and large-for-gestational-age (LGA) are defined as birth weights below the 10th percentile and above the 90th percentile, respectively [16]. Such deviations in fetal growth may result from a range of environmental and genetic factors, including maternal malnutrition, gestational comorbidities, smoking, alcohol consumption, and chromosomal abnormalities [18, 20, 21].

Because infants with abnormal birth weight may require specialized care for perinatal complications, parenting styles regarding feeding behavior often shift toward “catch-up” or “catch-down” growth management [18, 21]. Additionally, increased susceptibility to fetal hypoxic-ischemic events in these infants may contribute to enamel dysplasia or developmental enamel defects [14], both of which are established risk factors for ECC [3, 14]. Conversely, parents of these infants—particularly those born SGA—may adopt more rigorous health management practices, including stricter infection-control measures, because of perceived vulnerability [22, 23]. Given these complex interactions, the causal relationship between abnormal birth weight and ECC prevalence is multifaceted, involving various environmental and host factors. Consequently, a more comprehensive understanding of these socioeconomic and biological risk factors may facilitate the development of more effective strategies for ECC prevention.

Previous studies have reported an increased risk of ECC among children born SGA [3, 14, 24]. However, Tanaka et al. observed increased ECC prevalence among 3-year-old Japanese children born LGA, whereas no significant association was observed for SGA birth status [11, 25]. Similarly, a meta-analysis by Shi et al. reported that while preterm birth significantly increased ECC risk, low birth weight alone was not identified as an independent risk factor [12]. The Japan Environment and Children’s Study (JECS), a nationwide prospective cohort study, has collected data from approximately 100,000 mother–child dyads. This dataset includes information on ECC prevalence alongside maternal characteristics (e.g., age at delivery, smoking status, and alcohol intake), child characteristics (e.g., sex and congenital conditions), and oral health behaviors (e.g., toothbrushing frequency and fluoride use). Using this large-scale dataset, this study aimed to investigate the impact of birth weight for gestational age—specifically SGA and LGA—on ECC experiences through a secondary data analysis.

## Methods

### Data collection and participants

This birth cohort study was conducted in accordance with the principles of the Declaration of Helsinki and its subsequent revisions. This study used data obtained from the JECS, an ongoing nationwide prospective birth cohort study. The JECS protocol [26, 27] was reviewed and approved by the Institutional Review Board on Epidemiological Studies of the Ministry of the Environment, Japan, and the ethics committees of all participating institutions (approval no. 100910001).

Briefly, pregnant women visiting cooperating healthcare providers and local government offices between January 2011 and March 2014 were informed about the study objectives and procedures during early pregnancy. Written informed consent was obtained from all the participants before enrollment. Participants then completed self-administered questionnaires and underwent medical assessments conducted by physicians, midwives/nurses, and/or research coordinators at delivery. Early childhood data were collected semi-annually during the first 6 years postpartum through follow-up questionnaires. Details regarding data processing, validation, and verification of the perinatal assessment have been previously described [26, 27].

A total of 104,043 fetal records from 15 regional centers were initially enrolled in the JECS. Of these, 3,921 records (3.8%) were excluded because of miscarriage, stillbirth, or loss to follow-up. An additional 840 mother–child dyads were excluded because birth weight for gestational age could not be calculated, primarily due to the lack of available Japanese reference values for infants born before 22 weeks or after 42 weeks of gestation. Furthermore, 2,003 participants were excluded because of missing data on key covariates, including parity and infant birth weight. Among the remaining 97,279 mother–infant pairs, 18,273 lacked complete information on dental caries status during the first 5 years postpartum and were therefore excluded. The final analytical sample comprised 79,006 children (Fig. 1).

**Fig. 1:**
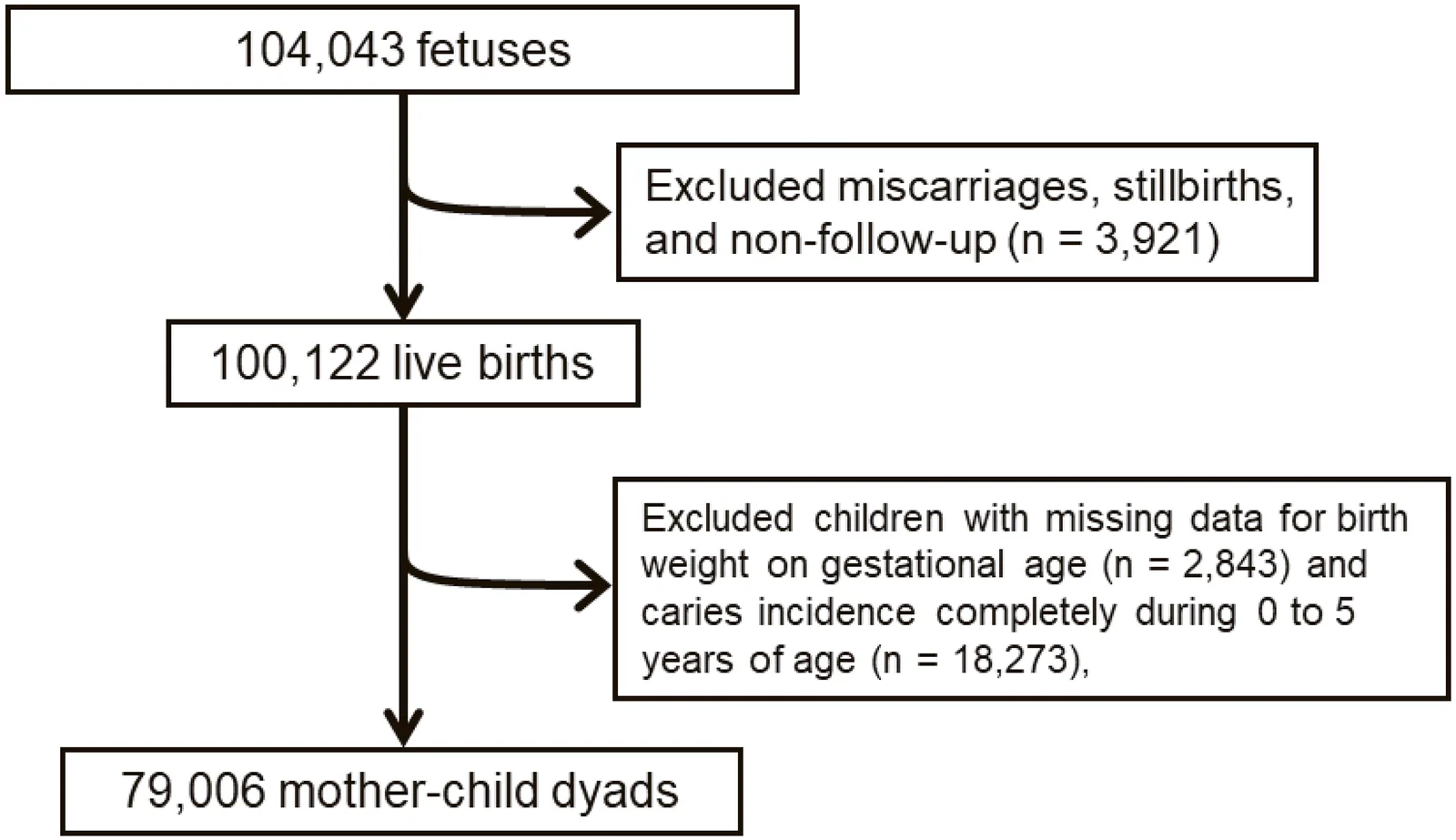
Flowchart of study participant selection.

### Dental caries experience: Outcomes

Information on ECC experience was collected through self-reported questionnaires administered at 4 and 5 years postpartum [26]. In the 4-year questionnaire, parents or caregivers reported whether their child had been diagnosed with dental caries at each age from 0 to 4 years. The 5.5-year questionnaire collected information on caries experience up to 5 years of age. For all analyses, ECC experience was treated as a binary variable (presence or absence). Based on the International Association of Paediatric Dentistry criteria [1], ECC was defined as any dental caries experience occurring between ages 0 and 5 years, while S-ECC was defined as any caries experience by 3 years of age. In the longitudinal analyses, participants who were reported to have dental caries at any assessment were classified as having ECC or S-ECC, as appropriate, at all subsequent time points.

For the main analysis, a generalized linear mixed model (GLMM) was employed to evaluate the longitudinal association of SGA and LGA with S-ECC and ECC experience. To assess the robustness of the findings to missing data, standard Poisson regression models were performed as cross-sectional sensitivity analyses using a multiply imputed dataset. These analyses specifically examined ECC experience at ages 3 years (S-ECC) and 5 years (ECC).

### Birth weight on gestational age: Exposure

Birth weight percentiles for gestational age were calculated using birth weight, gestational age, infant sex, and parity based on the standardized reference program provided by the Japanese Society for Pediatric Endocrinology (2010 edition; accessed July 7, 2025, at http://jspe.umin.jp/medical/keisan.html) [28, 29]. According to these reference values, children were classified into three categories: SGA (birth weight <10th percentile), LGA (birth weight >90th percentile), and appropriate-for-gestational-age (AGA; birth weight between the 10th and 90th percentiles), which served as the reference category in our analysis.

### Covariates

The questionnaire design has been previously described in detail [26, 27, 30]. Briefly, sociodemographic, lifestyle, and health-related characteristics of mother–child dyads, which are recognized as potential confounders of dental caries incidence in children [31–33], were included as covariates in the GLMMs. Data on maternal age at delivery, infant sex, gestational age, birth weight, and the presence of congenital diseases were retrieved from medical records and verified by doctors, midwives/nurses, and/or research coordinators. Information on parity, annual household income, smoking status, and alcohol consumption was collected through self-administered questionnaires completed by participating mothers during pregnancy. Data on oral care behaviors, including daily toothbrushing frequency, parental supervised toothbrushing behavior, and fluoride toothpaste use at 5 years postpartum, were obtained from the semi-annual follow-up questionnaires administered after delivery.

Participants were categorized according to the following variables: child sex (male or female); parity (primiparous or multiparous); annual household income in Japanese yen (<2, 2–<4, 4–<6, or ≥6 million yen); maternal educational attainment (junior/high school, junior college/technical college, or university/graduate school); maternal smoking status (never smoked, stopped before or during pregnancy, or currently smoking); maternal alcohol consumption (never drank, stopped before or during pregnancy, or current drinker); other congenital disease in infants (absence or presence); daily toothbrushing frequency (twice or more per day, once per day, or less than once per day); parental supervised toothbrushing (always, sometimes, or never); and fluoride toothpaste use (use, non-use, or unknown). Maternal age at delivery was assessed as a continuous variable.

### Statistical analysis

Continuous variables are presented as means with standard deviations (SDs), and categorical variables are presented as frequencies and percentages (Table 1). The primary objective of this study was to examine the longitudinal association between birth weight categories and ECC experience using a multilevel Poisson regression model within a GLMM framework. The GLMM provides a flexible and robust approach for analyzing longitudinal data, as it effectively handles missing data [34]. Given the binary nature of the outcome data, a Poisson distribution with a log-link function was applied to estimate risk ratios (RRs). To examine the longitudinal association, we utilized a long-format dataset in which the outcome was defined as the presence or absence of ECC at each follow-up assessment (ages 1, 2, 3, 4, and 5 years). Within-subject correlation across repeated measurements was accounted for by specifying a first-order autoregressive covariance structure.

**Table 1:** Basic characteristics on 79,006 mother-child pairs in the JECS.

	**AGA (n = 65,052)**	**SGA (n = 6,174)**	**LGA (n = 7,780)**
*Birth weight, Mean (SD)*	2997.2 (334.9)	2,376.2 (361.6)	3,638.1 (304.8)
*Birth weeks, Mean (SD)*	38.8 (1.6)	38.5(2.1)	38.9 (1.5)
*Maternal age, Mean (SD)*	31.4 (4.9)	31.6 (5.0)	31.6 (5.1)
missing	0 (0.0)	0 (0.0)	0 (0.0)
*Child’s sex, n (%)*			
boys	33,176 (51.0)	3,115 (50.5)	4,156 (53.4)
girls	31,876 (49.0)	3,059 (49.5)	3,624 (46.6)
missing	0 (0.0)	0 (0.0)	0 (0.0)
*Parity, n (%)*			
primiparae	26,659 (41.0)	2,685 (43.5)	3,553 (45.7)
multiparae	38,391 (59.0)	3,489 (56.5)	4,227 (54.3)
missing	2 (0.0)	0 (0.0)	0 (0.0)
*Household income (million yen/year), n (%)*			
<2	2,983 (4.6)	318 (5.2)	367 (4.7)
2 to <4	20,398 (31.4)	1,921 (31.1)	2,376 (30.5)
4 to <6	20,394 (31.4)	1,854 (30.0)	2,404 (30.9)
≥6	16,769 (25.8)	1,571 (25.4)	2,063 (26.5)
missing	4,508 (6.9)	510 (8.3)	570 (7.3)
*Smoking habit, n (%)*			
never	22,018 (33.8)	2,135 (34.6)	2,605 (33.5)
stopped	41,051 (63.1)	3,826 (62.0)	4,943 (63.5)
smoking	1,844 (2.8)	202 (3.3)	208 (2.7)
missing	139 (0.3)	11 (0.1)	24 (0.3)
*Alcohol consumption habits, n (%)*			
never	38,988 (59.9)	3,711 (60.1)	4,483 (57.6)
stopped	23,491 (36.1)	2,059 (33.3)	3,097 (39.8)
drinking	2,348 (3.6)	385 (6.2)	173 (2.2)
missing	225 (0.4)	19 (0.4)	27 (0.4)
*Educational attainment, n (%)*			
high school or less	21,550 (33.1)	2,133 (34.5)	2,722 (35.0)
junior college	27,820 (42.8)	2,613 (42.3)	3,233 (41.6)
university or higher	14,964 (23.0)	1,338 (21.7)	1,737 (22.3)
missing	718 (1.1)	90 (1.5)	88 (1.1)
*Presence of congenital diseases, n (%)*			
absence	62,113 (95.5)	5,679 (92.0)	7,423 (95.4)
presence	2,939 (4.5)	495 (8.0)	357 (4.6)
missing	0 (0.0)	0 (0.0)	0 (0.0)
*Daily brushing frequency, n (%)*			
twice or more	49,088 (75.5)	4,667 (75.6)	5,798 (74.5)
once	10,432 (16.0)	1,000 (16.2)	1,314 (16.9)
less than once	92 (0.1)	10 (0.2)	6 (0.1)
missing	5,440 (8.4)	497 (8.0)	662 (8.5)
*Parental supervised brushing, n (%)*			
always	42,119 (64.8)	4,045 (65.5)	4,845 (62.3)
sometimes	15,707 (24.1)	1,454 (23.6)	2,037 (26.2)
never	1,470 (2.3)	156 (2.5)	200 (2.6)
missing	5,756 (8.8)	519 (8.4)	698 (9.0)
*Fluoride toothpaste, n (%)*			
use	47,023 (72.3)	4,400 (71.2)	5,669 (72.9)
non-use	2,466 (3.8)	246 (4.0)	276 (3.5)
unknown	1,767 (2.7)	170 (2.8)	236 (3.0)
missing	13,796 (21.2)	1,358 (22.0)	1,599 (20.6)

Abbreviations: (A/L/S) GA, (appropriate/large/small) for gestational age; IQR, interquartile range; SD, standard deviation.

Sensitivity analyses were conducted using standard Poisson regression models applied to a wide-format dataset to evaluate the association between birth weight status and ECC at ages 3 and 5 years. These models employed the same distribution and link functions as the primary analysis but incorporated a robust Huber–White sandwich estimator to ensure reliable standard error estimates. To address missing data under the “missing-at-random” assumption, multiple imputation by chained equations was performed [35]. Ten imputed datasets were generated using linear and logistic regression models for covariates. Parameters were then pooled across these datasets according to Rubin’s rules [36] to assess the robustness of the longitudinal findings.

Both crude and multivariate models were fitted to estimate the RRs of the ECC experience, with the AGA group serving as the reference. Model 1 was adjusted for maternal age at delivery, parity, and child sex. Model 2 further included maternal educational attainment, smoking and drinking habits, household income, and the presence of congenital diseases. Model 3 further adjusted for oral health–related behaviors, including daily toothbrushing frequency, parental-supervised toothbrushing, and the use of fluoride toothpaste. An intercept was included as a random effect to account for between-individual baseline variability. Statistical significance was defined using 95% confidence intervals (CIs), and a sequential Bonferroni correction was applied to adjust for multiple comparisons of the estimated marginal means. All statistical analyses were performed using SPSS software (version 24.0; IBM Corp., Armonk, NY, USA).

## Results

Table 1 summarizes the baseline characteristics of participants according to birth weight for gestational age. Among the 79,006 participants included in the final analysis, 6,174 (7.8%) were classified as SGA, and 7,780 (9.8%) as LGA. Mean birth weight was 2376.2 g (SD 361.6) in the SGA group and 3638.1 g (SD 304.8) in the LGA group. The prevalence of congenital disease was higher in the SGA group (8.0%) than in the AGA (4.5%) and LGA (4.6%) groups. In contrast, other demographic characteristics, particularly oral care behaviors at 5 years of age, were generally similar across the groups. In particular, regarding oral care behaviors at 5 years of age, no substantial differences were observed among the three groups in the frequency of daily toothbrushing, parental assistance with toothbrushing, or the use of fluoride toothpaste.

Figure 2 presents the prevalence of ECC experience from 0 to 5 years of age, stratified by birth weight status (AGA, SGA, and LGA). The prevalence of ECC experience increased steadily with age in all three groups. By age 5 years, the prevalence of ECC experience reached approximately 48.0% in the LGA group, 43.9% in the AGA group, and 41.3% in the SGA group. Compared with the AGA group, children in the LGA group consistently exhibited a higher prevalence of ECC experience throughout the follow-up period. Conversely, those in the SGA group consistently exhibited a lower prevalence than the AGA group. Table S1 presents the raw data and corresponding 95% CIs for Fig. 2.

**Fig. 2:**
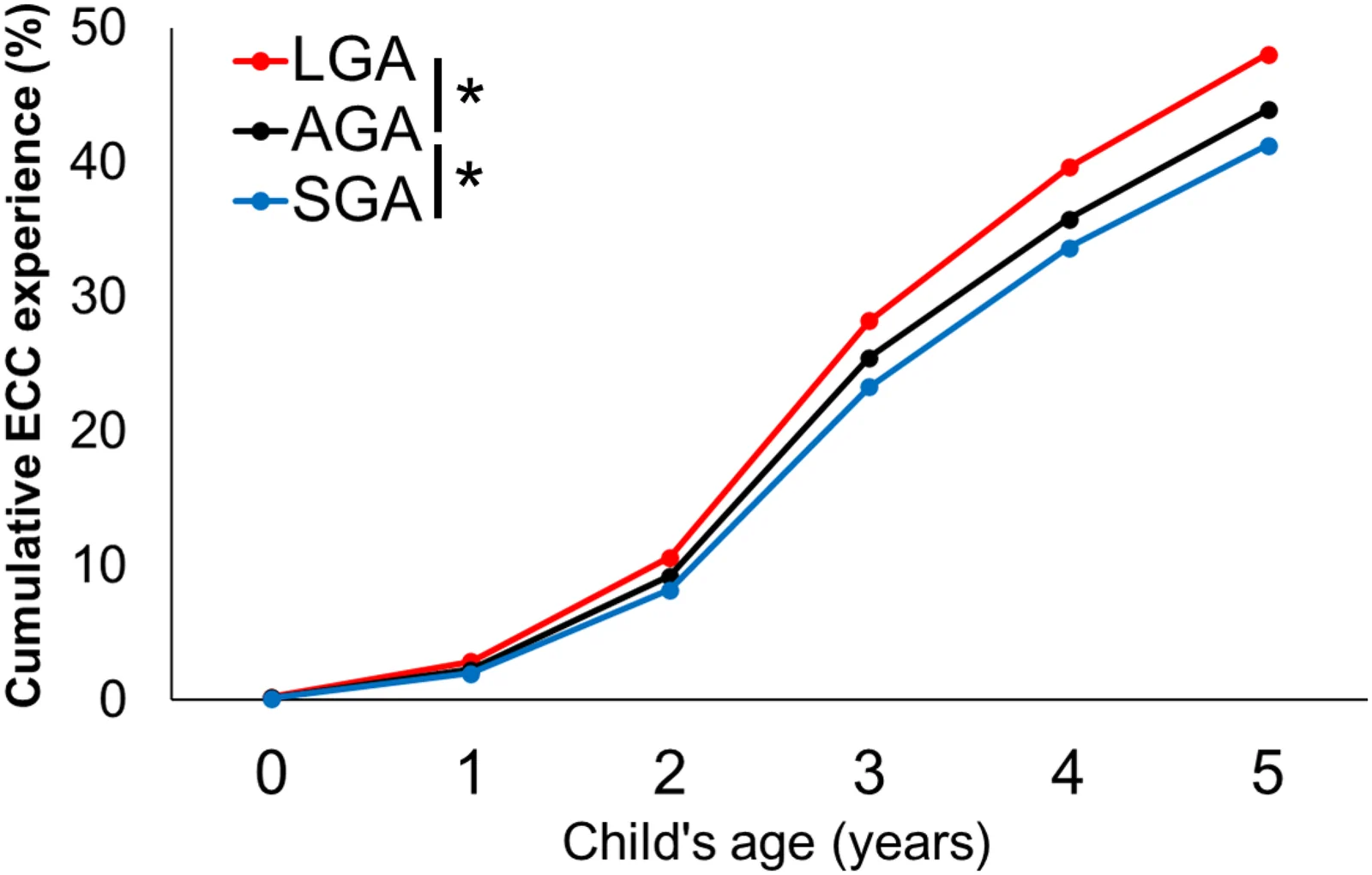
Longitudinal trajectories of cumulative ECC experience according to birth weight for gestational age. The lines represent the cumulative prevalence (%) of dental caries experience among children born large-for-gestational-age (LGA: red line), appropriate-for-gestational-age (AGA: black line), and small-for-gestational-age (SGA: blue line). Significant differences in the trajectories were observed among the three groups, with the LGA group consistently exhibiting the highest prevalence and the SGA group the lowest throughout the follow-up period (*p < 0.001 vs. AGA group, by GLMM). ECC, early childhood caries; GLMM, generalized linear mixed model.

Table 2 presents crude and adjusted RRs (with 95% CIs) for S-ECC and ECC experience based on birth weight status. The estimated RRs and their statistical significance remained consistently stable across all models. In the fully adjusted model (Model 3), the risk of both S-ECC and ECC was significantly higher in the LGA group (RR: 1.13, 95% CI: 1.08–1.18 for S-ECC; RR: 1.13, 95% CI: 1.10–1.15 for ECC) compared with the AGA group. In contrast, the SGA group exhibited a significantly lower risk of both outcomes (RR: 0.91, 95% CI: 0.86–0.96 for S-ECC; RR: 0.94, 95% CI: 0.92–0.97 for ECC). Consistent with these findings, sensitivity analyses using Poisson regression models with multiple imputation yielded similar estimates, further supporting the robustness of these associations (Tables S2 and S3).

**Table 2:** Association between birth weight for gestational age and early childhood caries experience.

	**AGA**	**SGA**	***p*-value**	**LGA**	***p*-value**
***S-ECC during birth to the age of 3 years***					
*Crude*	Ref	0.90 (0.87–0.94)	*<0.001*	1.13 (1.09–1.17)	*<0.001*
*Model 1*		0.92 (0.88–0.96)	*<0.001*	1.15 (1.11–1.19)	*<0.001*
*Model 2*		0.91 (0.87–0.95)	*<0.001*	1.13 (1.09–1.17)	*<0.001*
*Model 3*		0.91 (0.86–0.96)	*<0.001*	1.13 (1.08–1.18)	*<0.001*

***ECC during birth to the age of 5 years***					
*Crude*	Ref	0.93 (0.91–0.95)	*<0.001*	1.11 (1.09–1.13)	*<0.001*
*Model 1*		0.94 (0.92–0.96)	*<0.001*	1.13 (1.11–1.15)	*<0.001*
*Model 2*		0.94 (0.91–0.96)	*<0.001*	1.12 (1.10–1.14)	*<0.001*
*Model 3*		0.94 (0.92–0.97)	*<0.001*	1.13 (1.10–1.15)	*<0.001*

**Note:** Data are presented as risk ratios (95% confidence interval).

**Abbreviations:** (A/L/S) GA, (appropriate/large/small) for gestational age; GLMM, generalized linear mixed model; (S-)ECC, (severe) early childhood caries.

^a^Adjusted for maternal age at birth, parity status and child’s sex.

^b^Additionally adjusted for maternal educational attainment, smoking and drinking habit, household income, and infant’s prevalence of other congenital disease[s] with Model 1.

^c^Additionally adjusted for daily frequency of child’s toothbrushing, parental-supervised toothbrushing, and fluoride toothpaste with Model 2.

## Discussion

This study examined the association between birth weight for gestational age and ECC experience using a nationwide prospective birth cohort from Japan. The principal finding reveals a clear contrast: LGA was significantly associated with an increased risk of ECC, whereas SGA appeared to confer a protective association. These associations remained robust even after adjusting for socioeconomic status and oral health behaviors, underscoring the importance of understanding early-life risk factors for pediatric oral health in the prevention and management of ECC.

Although both SGA and LGA have been associated with a higher prevalence of enamel dysplasia [3, 14], the divergent patterns of ECC experience observed between these groups are particularly noteworthy. Translational rodent models have demonstrated that maternal nutritional restriction or gestational complications can alter odontogenesis, salivary gland development, and oral microbiota in offspring. Since ECC is caused by a complex interplay of host, socioeconomic, and microbiological factors, human epidemiological data remain scarce. Thus, future translational research is warranted to fully elucidate the precise biological pathways linking abnormal birth weight to altered dental development and increased ECC susceptibility. Consistent with previous observations [3, 11–15], LGA was associated with an increased risk of ECC. Because LGA is often linked to maternal gestational diabetes and excessive gestational weight gain [15], LGA infants face increased risks of fetal hypoxia-ischemia, neonatal hypoglycemia, and subsequent childhood metabolic syndrome [15]. This chronic gestational complicated state during the critical prenatal period of tooth development can result in enamel dysplasia [13, 37], which in turn provides the elevated susceptibility to ECC observed in our study. Evidently, it is relevant to note that maternal obesity is also associated with poorer oral health, including increased risks of dental caries and tooth loss [38, 39]. The prevalence of obesity among LGA babies progressively increases during early childhood, reaching approximately one-third by age 3 years [15, 40], potentially reflecting postnatal dietary patterns characteristic of accelerated growth trajectories [41]. Supporting this possibility, Deng et al. [42] reported that adolescents with obesity exhibit significantly higher salivary *Streptococcus mutans* levels and lower stimulated salivary flow rates than lean controls. Collectively, these findings suggest that the increased ECC risk in LGA children may be mediated by altered salivary composition, early-life metabolic programming, and dietary patterns in early childhood that promote a sugar-rich, biofilm-driven cariogenic environment. Since we previously demonstrated that habitual parental supervised brushing reduces the risk of ECC by approximately 25% [43], early preventive approaches for LGA children must prioritize dietary guidance alongside habitual parental management with fluoride applications.

In contrast, the lower ECC risk observed in the SGA group suggests that biological vulnerabilities, such as enamel hypoplasia resulting from fetal growth restriction, do not necessarily translate into higher caries experience [3, 14]. In SGA infants, well-documented non-dental health consequences include neonatal asphyxia and long-term neurodevelopmental delays [44, 45], which often arise from gestational hypoxic-ischemia events and maternal-fetal nutritional deprivation. These prenatal insults directly predispose primary teeth to severe enamel hypoplasia [3]. Although our epidemiological findings indicate a lower cumulative ECC prevalence in SGA children—potentially offset by the heightened parental monitoring and stricter feeding practices discussed below—their underlying biological vulnerability to enamel hypoplasia remains a critical risk factor that warrants early preventive screening. Fernandes et al. [3] proposed that the timing of primary tooth eruption may strongly influence ECC incidence in SGA children; however, first tooth eruption has been reported to be delayed in both SGA [46] and LGA [37] infants, which may partially mitigate early exposure to cariogenic risk.

Regarding dietary behavior, SGA children often require nutritional catch-up growth during early childhood [44], while mothers of SGA children more commonly have lower body mass index due to suboptimal dietary intake [45]. In addition, parents of SGA infants, perceiving their children as more vulnerable due to neonatal morbidity or infection risks, may adopt stricter health and infection control, including more vigilant feeding practices [47–49]. Since our findings align with the hypothesis that parental health management plays a crucial role, heightened parental monitoring in SGA children may inadvertently serve as a primary intervention, offsetting biological susceptibilities such as enamel defects, at least in early childhood. However, regarding oral care behaviors at 5 years of age (Table 1), no substantial differences were observed among the three groups. Furthermore, the observed 20–25% difference in RRs between the LGA and SGA groups suggests that birth weight status may serve as a valuable index for identifying high-risk children during pediatric dental checkups. Effect size estimates based on cumulative ECC incidence in the AGA group at 5 years were small (−0.06 for SGA and 0.10 for LGA), indicating modest effect magnitudes. Nevertheless, our findings do not preclude the need for tailored preventive strategies, including early dietary counseling and intensified fluoride exposure, particularly for SGA children who may still be vulnerable to caries development due to enamel dysplasia.

Despite the strengths of this study, including its large-scale prospective design and adjustment for multiple socioeconomic and behavioral factors, several limitations should be acknowledged. First, ECC and S-ECC were assessed using parent-reported questionnaires rather than clinical dental examinations. Although self-reported oral health data from the JECS have been summarized and validated in previous studies [26, 27, 49], reporting bias and potential underestimation of caries experience—particularly in its early stages—cannot be excluded. Second, despite adjustment for maternal smoking, alcohol consumption, and household income, residual confounding may persist. The JECS dataset did not include detailed dietary information, such as the frequency of sugar intake or the duration of nocturnal breastfeeding, both of which are established contributors to ECC etiology [1]. The absence of these variables may have influenced the observed associations. Accordingly, further studies are needed to elucidate the mechanisms underlying the observed lower risk of ECC among SGA children. Finally, although the JECS is a nationwide cohort, participation was voluntary, which may introduce healthy volunteer bias. Families with greater health awareness may have been more likely to enroll and remain in the study, potentially resulting in lower observed ECC prevalence than in the general Japanese population.

## Conclusions

Children born SGA were prospectively associated with a lower prevalence of ECC up to 5 years of age, whereas those born LGA exhibited a significantly higher prevalence. These contrasting associations underscore birth weight for gestational age as an important risk indicator for ECC. A deeper understanding of the socioeconomic and biological mechanisms underlying these relationships may inform the development of more targeted preventive and curative management strategies for ECC.

## Abbreviations

AGA: appropriate for gestational age
CI: confidence interval
GLMM: generalized linear mixed model
IQR: interquartile range
JECS: Japan Environment and Children’s Study
LGA: large for gestational age
Ref: reference
RR: risk ratio
SD: standard deviation
(S-)ECC: (severe) early childhood caries
SGA: small for gestational age

## Supplementary information

**Additional file 1:** **Table S1.** Prevalence of ECC experience from birth to 5 years of age.

**Additional file 2:** **Table S2.** Basic characteristics of 79,006 mother–child pairs in the JECS after multiple imputation.

**Additional file 3:** **Table S3.** Results of standard Poisson regression models assessing the associations between birth weight for gestational age and ECC experience at 3 and 5 years of age.

## Declarations

### Availability of data and materials

Data are unsuitable for public deposition due to ethical restrictions and legal framework of Japan. It is prohibited by the Act on the Protection of Personal Information (Act No. 57 of 30 May 2003, amendment on 9 September 2015) to publicly deposit the data containing personal information. Ethical Guidelines for Medical and Health Research Involving Human Subjects enforced by the Japan Ministry of Education, Culture, Sports, Science and Technology and the Ministry of Health, Labour and Welfare also restricts the open sharing of the epidemiologic data. All inquiries about access to data should be sent to: jecs-en@nies.go.jp. The person responsible for handling enquiries sent to this e-mail address is Dr Shoji F. Nakayama, JECS Programme Office, National Institute for Environmental Studies.
