# Supplementary material for: Dental caries incidence in infants with abnormal birth weight for gestational age: a longitudinal study in the Japan Environment and Children’s Study
Source: Environ Health Prev Med. 2026 Sep 30;31:66. doi: 10.1265/ehpm.26-00133 (PMC13649617; doi:10.1265/ehpm.26-00133)
Supplement: Supplementary file 1 [file ehpm-31-066-s001.pdf]

Table S1. Prevalence of ECC experience from birth to 5 years of age.

	Age (years)					
	**0**	**1**	**2**	**3**	**4**	**5**
**LGA (n = 7,780)**	18 (0.2 [0.2–0.2])	215 (2.9 [2.9–2.9])	795 (10.6 [10.5–10.7])	2,110 (28.2 [28.0–28.4])	2,965 (39.6 [39.3–39.9])	3,592 (48.0 [47.6–48.4])
**AGA (n = 65,052)**	120 (0.2 [0.2–0.2])	1,403 (2.2 [2.2–2.2])	5,751 (9.2 [9.1–9.3])	15,903 (25.5 [25.2–25.8])	22,321 (35.8 [35.4–36.2])	27,399 (43.9 [43.4–44.4])
**SGA (n = 6,174)**	8 (0.1 [0.1–0.1])	117 (2.2 [2.2–2.2])	487 (8.2 [8.1–8.3])	1,385 (23.3 [23.0–23.6])	2,001 (33.6 [33.3–33.9])	2,455 (41.3 [40.9–41.7])

Note: Data are presented as number (percentage [95% confidence interval]).

Abbreviations: (A/L/S) GA, (appropriate/large/small) for gestational age; CI, confidence interval; ECC, early childhood caries.
