# Supplementary material for: Dental caries incidence in infants with abnormal birth weight for gestational age: a longitudinal study in the Japan Environment and Children’s Study
Source: Environ Health Prev Med. 2026 Sep 30;31:66. doi: 10.1265/ehpm.26-00133 (PMC13649617; doi:10.1265/ehpm.26-00133)
Supplement: Supplementary file 2 [file ehpm-31-066-s002.pdf]

Table S2. Basic characteristics on 79,006 mother-child pairs in the JECS after multiple imputation.

	AGA (n = 65,052)	SGA (n = 6,174)	LGA (n = 7,780)
Birth weight, Mean (SD)	2997.2 (334.9)	2,376.2 (361.6)	3,638.1 (304.8)
Birth weeks, Mean (SD)	38.8 (1.6)	38.5(2.1)	38.9 (1.5)
Maternal age, Mean (SD)	31.4 (4.9)	31.6 (5.0)	31.6 (5.1)
Child's sex, n (%)			
Boys	33,176 (51.0)	3,115 (50.5)	4,156 (53.4)
Girls	31,876 (49.0)	3,059 (49.5)	3,624 (46.6)
Parity, n (%)			
Primiparae	26,659 (41.0)	2,685 (43.5)	3,553 (45.7)
Multiparae	38,393 (59.0)	3,489 (56.5)	4,227 (54.3)
Household income (million yen/ year), n (%)			
<2	3,653 (5.6)	389 (6.3)	448 (5.8)
2 to <4	21,581 (33.2)	2,052 (33.2)	2,530 (32.5)
4 to <6	21,849 (33.6)	2,018 (32.7)	2,592 (33.3)
≥6	17,969 (27.6)	1,716 (27.8)	2,210 (28.4)
Smoking habit, n (%)			
Never	39,116 (60.1)	3,722 (60.3)	4,496 (57.8)
Stopped	23,578 (36.2)	2,066 (33.5)	3,110 (40.0)
Smoking	2,358 (3.6)	386 (6.3)	174 (2.2)
Alcohol consumption habits, n (%)			
Never	22,064 (33.9)	2,138 (34.6)	2,613 (33.6)
Stopped	41,140 (63.2)	3,834 (62.1)	4,959 (63.7)
Drinking	1,848 (2.8)	202 (3.3)	208 (2.7)
Educational attainment, n (%)			
High school or less	21,719 (33.4)	2,153 (34.9)	2,740 (35.2)
Junior college	28,262 (43.4)	2,668 (43.2)	3,290 (42.3)
University or higher	15,072 (23.2)	1,353 (21.9)	1,751 (22.5)
Presence of congenital diseases, n (%)			
Absence	62,113 (95.5)	5,679 (92.0)	7,423 (95.4)
Presence	2,939 (4.5)	495 (8.0)	357 (4.6)
Daily brushing feaquency, n (%)			
Twice or more	53,528 (82.3)	5,078 (82.2)	6,335 (81.4)
Once	11,363 (17.5)	1,084 (17.6)	1,427 (18.3)
Less than once	161 (0.2)	12 (0.2)	18 (0.2)
Parental supervised brushing, n (%)			
Always	45,973 (70.7)	4,410 (71.4)	5,307 (68.2)
Sometimes	17,319 (26.6)	1,587 (25.7)	2,236 (28.7)
Never	1,761 (2.7)	177 (2.9)	237 (3.0)
Fluoride toothpaste, n (%)			
Use	59,600 (91.6)	5,637 (91.3)	7,126 (91.6)
Non-use	3,186 (4.9)	321 (5.2)	355 (4.6)
Unknown	2,267 (3.5)	216 (3.5)	299 (3.8)

Abbreviation: (A/L/S) GA, (appropriate/large/small) for gestational age; IQR, interquartile range; SD, standard deviation.
The totals may not equal the sum of the individual counts due to multiple imputation, with rounding performed based on a standard cutoff of 0.5.
