# Supplementary material for: Dental caries incidence in infants with abnormal birth weight for gestational age: a longitudinal study in the Japan Environment and Children’s Study
Source: Environ Health Prev Med. 2026 Sep 30;31:66. doi: 10.1265/ehpm.26-00133 (PMC13649617; doi:10.1265/ehpm.26-00133)
Supplement: Supplementary file 3 [file ehpm-31-066-s003.pdf]

Table S3. Results of standard Poisson regression models assessing the associations between birth weight for gestational age and ECC experience at 3 and 5 years of age.

	AGA	SGA	*p*-value	LGA	*p*-value
**S-ECC experience at 3 years-old, n (%)**	17,592 (27.0%)	1,531 (24.8%)		2,301 (29.6%)	
Crude	Ref	0.92 (0.88–0.96)	*<0.001*	1.09 (1.05–1.14)	*<0.001*
Model 1		0.93 (0.89–0.97)	*0.001*	1.12 (1.07–1.16)	*<0.001*
Model 2		0.91 (0.87–0.96)	*<0.001*	1.11 (1.07–1.15)	*<0.001*
Model 3		0.92 (0.88–0.96)	*<0.001*	1.10 (1.06–1.15)	*<0.001*
	AGA	SGA	*p*-value	LGA	*p*-value
**ECC experience at 5 years-old, n (%)**	27,399 (42.1%)	2,455 (39.8%)		3,592 (46.2%)	
Crude	Ref	0.94 (0.91–0.97)	*<0.001*	1.10 (1.07–1.12)	*<0.001*
Model 1		0.95 (0.92–0.98)	*<0.001*	1.11 (1.08–1.14)	*<0.001*
Model 2		0.94 (0.91–0.97)	*<0.001*	1.11 (1.08–1.13)	*<0.001*
Model 3		0.94 (0.91–0.97)	*<0.001*	1.10 (1.07–1.13)	*<0.001*

Note: Data are presented as risk ratios (95% confidence intervals).

Abbreviations: (A/L/S) GA, (appropriate/large/small) for gestational age; (S-)ECC, (severe) early childhood caries.

[a]Adjusted for maternal age at birth, parity status and child's sex.

[b]Additionally adjusted for maternal educational attainment, smoking and drinking habits, household income, and infant's prevalence of other congenital disease[s] with Model 1.

[c]Additionally adjusted for daily frequency of child's toothbrushing, parental-supervised toothbrushing, and fluoride toothpaste with Model 2.
